# The Proinflammatory Soluble CD40 Ligand Is Associated with the Systemic Extent of Stable Atherosclerosis

**DOI:** 10.3390/medicina57010039

**Published:** 2021-01-04

**Authors:** Tiago Pereira-da-Silva, Patrícia Napoleão, Teresa Pinheiro, Mafalda Selas, Filipa Silva, Rui Cruz Ferreira, Miguel Mota Carmo

**Affiliations:** 1 Department of Cardiology, Hospital de Santa Marta, Centro Hospitalar Universitário de Lisboa Central, 1169-024 Lisbon, Portugal; mafalda.selas@gmail.com (M.S.); felipafernandes@gmail.com (F.S.); cruzferreira@netcabo.pt (R.C.F.); 2 NOVA Medical School, Faculdade de Ciências Médicas, Universidade NOVA de Lisboa, 1169-056 Lisbon, Portugal; 3 Instituto de Medicina Molecular João Lobo Antunes, Faculdade de Medicina, Universidade de Lisboa, 1649-028 Lisbon, Portugal; napoleao.patricia@gmail.com; 4 Instituto de Bioengenharia e Biociências, Departamento de Engenharia e Ciências Nucleares, Instituto Superior Técnico, Universidade de Lisboa, 2695-066 Lisbon, Portugal; teresa.pinheiro@tecnico.ulisboa.pt; 5 Chronic Diseases Research Center (CEDOC), NOVA Medical School, Faculdade de Ciências Médicas, Universidade NOVA de Lisboa, 1150-082 Lisbon, Portugal; mabmc@sapo.pt

**Keywords:** atherosclerosis, carotid artery disease, coronary artery disease, inflammation, lower extremity arterial disease, soluble CD40 ligand

## Abstract

*Background and objectives*: Polyvascular atherosclerosis is frequent and associated with a high cardiovascular risk, although the mechanisms regulating the atherosclerosis extent to single or multiple arterial territories are still poorly understood. Inflammation regulates atherogenesis and soluble CD40 ligand (sCD40L) is an inflammatory mediator associated with the presence of single-territorial atherosclerosis. We assessed whether the sCD40L expression is associated with the atherosclerosis extent to single or multiple arterial territories and with the atherosclerosis severity in different territories. *Materials and Methods*: We prospectively enrolled 94 participants with no atherosclerosis (controls, *n* = 26); isolated coronary atherosclerosis (group 1, *n* = 20); coronary and lower extremity (LE) atherosclerosis (group 2, *n* = 18); coronary and carotid atherosclerosis (group 3, *n* = 12); and coronary, LE, and carotid atherosclerosis (group 4, *n* = 18). Serum sCD40L levels were quantified. *Results:* The sCD40L levels (ng/mL, mean (standard deviation)) were 4.0 (1.5), 5.6 (2.6), 7.2 (4.2), 5.9 (3.7), and 5.1 (2.4) in controls and groups 1 to 4, respectively (ANOVA *p* = 0.012). In nonrevascularized patients, the sCD40L levels were significantly higher in group 2 than in group 1 and were correlated with the number of LE diseased segments. Prior LE bypass surgery was associated with lower sCD40L levels. Coexistence of coronary and LE atherosclerosis was independently associated with the sCD40L levels. *Conclusions*: The sCD40L levels were increased in stable atherosclerosis, particularly in polyvascular coronary and LE atherosclerosis. The number of LE diseased segments and prior LE revascularization were associated with sCD40L expression. To our knowledge, these are novel data, which provide insights into the mechanisms underlying multi-territorial atherosclerosis expression. sCD40L may be a promising noninvasive tool for refining the stratification of the systemic atherosclerotic burden.

## 1. Introduction

Little is known on the mechanisms that regulate the atherosclerosis extent to single or multiple arterial beds [1,2]. Specifically, it is not completely understood why some patients develop isolated coronary artery disease (CAD), while others develop a more systemic disease involving coronary and extra-coronary lesions [1,2]. Inflammation regulates atherogenesis and could partially explain the heterogeneity of stable atherosclerosis presentation [3]. The importance of identifying inflammatory mediators associated with stable atherosclerosis expression is recognized not only for better understanding of the pathophysiology, but also for clinical practice since they may potentially be used as diagnostic biomarkers and therapeutic targets [4]. In fact, the study on inflammation recently regained interest after the groundbreaking results of a pure anti-inflammatory agent in patients with atherosclerosis, in the Canakinumab Antiinflammatory Thrombosis Outcome Study [4].

The soluble CD40 ligand (sCD40L) is a proinflammatory mediator that has a mechanism of action distinct from other inflammatory mediators [5,6,7,8]. It is released into circulation mainly by activated platelets and interacts with different cells that participate in vascular inflammation and atherogenesis, including endothelial cells, macrophages, and T cells [5,6,7,8]. Furthermore, sCD40L is associated with vascular function [9,10]. Previous studies reported increased sCD40L levels in stable atherosclerosis of single territories, including the coronary [11,12,13], lower extremity (LE) [14,15,16], and carotid [17,18,19] vascular beds. To the best of our knowledge, there is no published prospective data on a potential association between sCD40L and disease extent to single or multiple arterial territories. Polyvascular atherosclerosis warrants special attention not only because it may have a different pathophysiology from that of single-vascular atherosclerosis, but also because it is frequent in clinical practice and associated with a higher risk of ischemic events [20,21,22]. On the other hand, the sCD40L levels were reported to be associated with CAD severity [12,13], whereas data on the sCD40L expression according to extra-coronary atherosclerosis severity is very scarce [23,24].

This pilot study aimed to explore whether the serum sCD40L levels are associated with the extent of stable atherosclerosis to single (coronary) or multiple (coronary and extra-coronary) arterial territories and with the atherosclerosis severity in extra-coronary territories. We hypothesized that the sCD40L levels differ according to the systemic extent of atherosclerosis, specifically in isolated CAD and polyvascular atherosclerosis, and the severity of atherosclerosis in different territories.

## 2. Materials and Methods

This study is a part of a project aimed at assessing the mechanisms underlying stable atherosclerosis expression in single (coronary) and multiple (coronary plus LE and/or carotid) arterial territories. The study protocol was approved by the ethics committees of the involved institutions (NOVA Medical School, Faculdade de Ciências Médicas, Universidade NOVA de Lisboa, Nr. 000176, in 11 November 2015, and Centro Hospitalar Universitário de Lisboa Central, Nr. 245/2015, in 1 October 2015). The investigation conformed to the principles outlined in the Declaration of Helsinki. All participants signed informed consent forms for inclusion before they participated in the study.

### 2.1. Recruitment of Participants

For assessing the sCD40L expression in atherosclerosis of single and multiple vascular beds, we prospectively recruited five groups of age- and sex-matched participants followed in our center: controls, with no coronary, LE, or carotid atherosclerosis; group 1, with isolated CAD; group 2, with coronary and LE atherosclerosis; group 3, with coronary and carotid atherosclerosis; and group 4, with atherosclerosis of the three territories. All participants were screened for obstructive atherosclerotic disease in the three territories. CAD was excluded in controls if they presented no effort angina; no evidence of CAD on coronary computed tomography angiography, including a calcium score of 0 and no soft plaques; and no positive myocardial stress test (the latter was not mandated to be assessed as per protocol). In other participants, CAD was defined as luminal stenosis of at least 50% for the left main artery or at least 70% for other epicardial vessels on invasive coronary angiography. LE arterial disease was defined as a significant (≥50%) stenosis on Doppler ultrasound (DUS) at rest [25,26] or the combination of chronic claudication and an ankle–brachial index equal to or less than 0.9 [26,27]. DUS was performed for the characterization of LE arterial disease. LE arterial disease was excluded in the case of no significant (≥50%) stenosis on DUS or in the case of absent chronic claudication and an ankle–brachial index higher than 0.9 [26,27]. Carotid artery disease was defined as stenosis of at least 50% on DUS and was excluded in the absence of any significant (≥50%) stenosis [26,27]. The mean and maximal intima–media thickness (IMT) were measured as previously described in individuals without overt arterial injury (in case of no significant carotid artery stenosis) [28]. All DUS studies of the LE and carotid arteries were performed according to a standardized protocol, using the GE Logiq S7 Expert Ultrasound System, and measurements were performed while following published guidelines [26,27,29].

The exclusion criteria were as follows: patients with acute ischemic events within 12 months, either coronary, LE, or cerebrovascular events; those with coronary artery bypass grafting (CABG) or LE bypass surgery performed within 12 months; those with prior carotid endarterectomy or prior percutaneous intervention of the coronary, LE, or carotid arteries; those with critical limb ischemia (with ischemic rest pain), heart failure, hemodynamically significant valvular heart disease, hematological disorders, active infection, history of malignancy, chronic kidney disease (stage 4 or 5), or severe hepatic dysfunction; those under 18 years of age; or those unable or unwilling to consent to study participation. If performed at least 12 months before inclusion, prior CABG and/or LE bypass surgery were not exclusion criteria since the presence and extent of atherosclerotic lesions, which were the focus of this study, are not modified by the surgical placement of bypass conduits [26,30].

### 2.2. Sample Size

No previous studies reported data on the sCD40L levels in both single- and multi-territorial atherosclerosis with the systematic assessment of different territories, which would be valuable for supporting the sample size estimation in our study. In this pilot study, we planned to recruit at least 20 controls, 20 patients with isolated CAD (group 1), and 40 patients with coronary and extra-coronary atherosclerosis (groups 2 to 4), including at least 10 patients per group in groups 2 to 4. The recruitment of participants for each group continued even after meeting the minimum number of participants until the minimum sample size was achieved for all groups.

### 2.3. Data Collection

Data were collected prospectively after patient inclusion. A standardized record of clinical, demographic, laboratory, echocardiographic, DUS, computed tomography angiography, and invasive coronary angiography data was obtained from each participant. For evaluating the severity of LE disease, the number of arterial segments with obstructive disease on both sides was assessed, including the external iliac, common femoral, superficial femoral, popliteal, anterior tibial, posterior tibial, and fibular arteries [25,26,31]. The LE lesions were classified as proximal if they were located in the popliteal artery or above or distal if they were located below [26].

### 2.4. Blood Sampling and sCD40L Measurements

Peripheral blood was collected early in the morning under fasting conditions. Serum was separated by centrifugation (500× *g* for 10 min) within 15 min of sampling. Aliquots were stored at −80 °C; samples were thawed only once. The sCD40L levels were measured in serum by an enzyme-linked immunosorbent assay commercial kit (R&D Systems, Minneapolis, MN, USA). Each sample was measured in duplicate. The intra-assay variation among the duplicates for all samples was less than 10%.

### 2.5. Statistical Analysis

Discrete variables are presented as frequency (percentage); continuous variables are presented as mean (standard deviation) in normally distributed data or median (interquartile range) in variables without a normal distribution (Shapiro–Wilk test). Categorical variables were analyzed using the chi-square or Fisher’s exact tests. Continuous variables were analyzed using Student’s t-test or the Mann–Whitney test when normality was not verified. Comparisons between multiple groups were performed using the analysis of variance (ANOVA) in normally distributed data and Kruskal–Wallis test in variables without a normal distribution; the Bonferroni post hoc correction was used for multiple pairwise comparisons. Pearson’s correlation was used to test correlations between continuous variables. The multivariate linear regression analysis was performed to identify the independent predictors of the sCD40L levels, among all available data. Outliers were excluded, as appropriate [32]. Considering the association between prior CABG or LE bypass surgery and lower sCD40L levels in our sample, we performed a post hoc analysis to explore whether the sCD40L levels varied with the atherosclerosis extent to single or multiple arterial territories and with the atherosclerosis severity in extra-coronary territories, excluding patients with prior revascularization. The level of significance considered was α = 0.05. Analyses were conducted using the SPSS software, version 26 (IBM).

## 3. Results

### 3.1. Clinical Characteristics, Laboratory Results, and Atherosclerosis Data of Participants

A total of 94 participants were included: 26 controls, 20 with isolated CAD (group 1), 18 with coronary and LE disease (group 2), 12 with coronary and carotid disease (group 3), and 18 with disease of the three territories (group 4). Clinical characteristics, laboratory results, and atherosclerosis data of participants are presented in Table 1. The differences in clinical characteristics and laboratory data across groups were driven by controls, where hypertension, dyslipidemia, diabetes mellitus, smoking history, and the use of antiplatelet and statin therapy were less prevalent; the neutrophil count, neutrophil/lymphocyte ratio, and creatinine levels were lower; and the high-density lipoprotein cholesterol levels were higher than those in patients with atherosclerosis. The distribution of these parameters did not differ across groups 1 to 4.

Regarding atherosclerosis data, the distribution of CAD parameters did not differ significantly across groups 1 to 4, including the number of vessels with obstructive disease, number of obstructive lesions, Gensini score, or rates of prior CABG.

Among patients with LE atherosclerosis (groups 2 and 4), group 4 showed a higher prevalence of bilateral LE disease and higher number of LE arterial segments with obstructive disease. The presence of proximal LE lesions and rates of prior LE bypass surgery did not differ between groups 2 and 4, although the revascularization rates were nonsignificantly higher in the latter (11.1% vs. 33.3%, respectively, *p* = 0.109).

Regarding patients with carotid artery disease (groups 3 and 4), there were no differences between the two groups regarding the rates of bilateral carotid artery disease. The maximal and mean IMT did not differ across controls and groups 1 and 2.

### 3.2. Variation of the sCD40L Levels According to the Systemic Extent of Atherosclerosis

**Figure 1:**
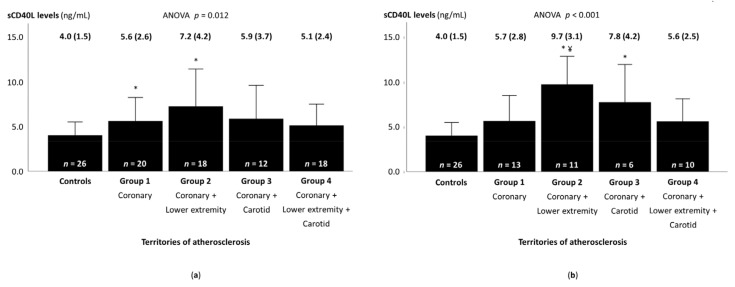
Soluble CD40 ligand levels according to the presence of atherosclerosis in different arterial territories. (**a**) in the whole sample, and (**b**) excluding patients with prior revascularization of the coronary and/or lower extremity arterial territories. Soluble CD40 ligand values are expressed as mean (standard deviation). sCD40L—soluble CD40 ligand. * *p*-Value < 0.05 vs. controls; ¥ *p*-Value < 0.05 vs. isolated coronary artery disease.

The sCD40L levels differed across groups (ANOVA p = 0.012) (Figure 1a). Patients from groups 1 (isolated CAD) and 2 (coronary and LE disease) showed significantly higher sCD40L levels than controls, and those from groups 3 (coronary and carotid disease) and 4 (disease of the three territories) showed nonsignificantly higher sCD40L levels than controls. Excluding patients with prior CABG and/or LE bypass surgery, the sCD40L levels were significantly higher in patients from group 2 (coronary and LE disease) compared with those in patients from group 1 (isolated CAD) (Figure 1b).

### 3.3. Variation of the sCD40L Levels According to the Severity of Atherosclerosis in Extra-Coronary Territories

**Figure 2:**
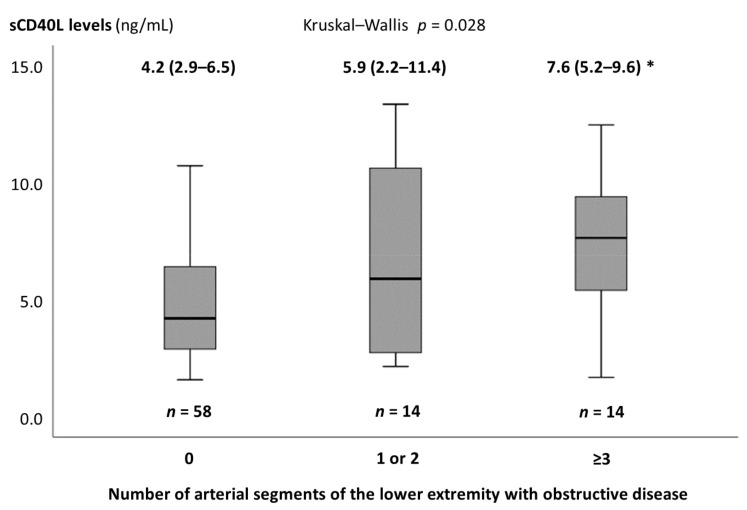
Soluble CD40 ligand levels according to the number of segments of the lower extremity with obstructive disease in patients without prior lower extremity bypass surgery. Soluble CD40 ligand values are expressed as median (interquartile range). sCD40L—soluble CD40 ligand. * *p*-Value < 0.05 vs. no segments with obstructive disease.

For the LE disease, no significant correlation was found between the sCD40L levels and number of arterial segments with obstructive disease (r = 0.157, *p* = 0.147). However, a weak positive correlation between the sCD40L levels and number of arterial segments with obstructive disease was observed excluding patients with prior LE bypass surgery (r = 0.238, *p* = 0.034) and those with prior CABG and/or LE bypass surgery (r = 0.281, *p* = 0.027). The median number of LE segments with obstructive disease was 3 (interquartile range 2–5); classifying patients with LE disease according to the median number of diseased segments, the sCD40L levels were significantly higher in patients with three or more LE diseased segments, but not in patients with less than three, compared with those with no LE disease (Figure 2). sCD40L did not differ according to the presence of bilateral or proximal LE disease (Supplementary Materials, Table S1).

**Figure 3:**
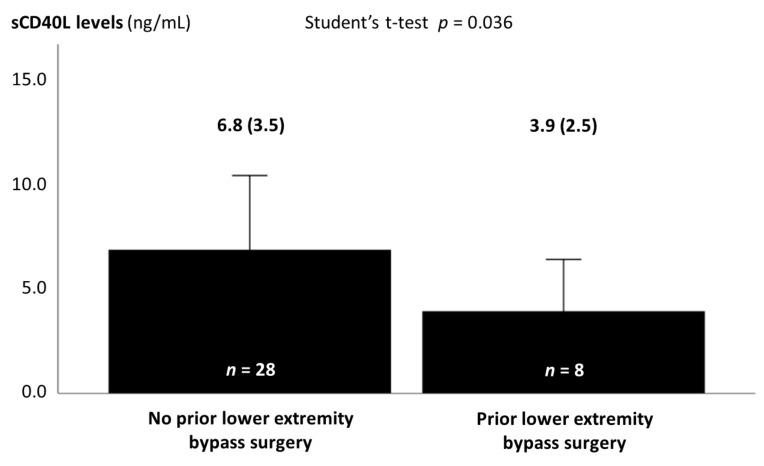
Soluble CD40 ligand levels in patients with lower extremity atherosclerosis with and without prior lower extremity bypass surgery. Soluble CD40 ligand values are expressed as mean (standard deviation). sCD40L—soluble CD40 ligand.

Of note, among patients with LE disease, prior LE bypass surgery was associated with lower sCD40L levels (Figure 3). The median time elapsed from LE bypass surgery was 4 years (interquartile range 2–9 years) (Supplementary Materials, Table S2). There were no differences between patients with and without prior LE bypass surgery regarding clinical characteristics, other laboratory data, CAD severity, rates of prior CABG, or proportion of bilateral or of proximal LE disease; there was a trend for a higher number of LE diseased segments in patients with prior LE bypass surgery (Supplementary Materials, Table S2).

For carotid artery disease, the sCD40L levels showed no association with the presence of bilateral disease and no correlation with the mean or maximal IMT (Supplementary Materials, Table S1). Furthermore, no associations were found after excluding patients with prior CABG and/or LE bypass surgery (data not shown).

### 3.4. Predictors of the sCD40L Levels

A detailed univariate analysis on parameters associated with the sCD40L levels is presented in Table S1 (Supplementary Materials) and a stratified analysis by study group is presented in Table S3 (Supplementary Materials). In the multivariate linear regression analysis, the independent predictors of the sCD40L levels were coexistent coronary and LE obstructive atherosclerosis, prior CABG, and leukocyte count (Table 2).

## 4. Discussion

In this prospective case–control study, three main findings stood out: the sCD40L levels varied according to the systemic extent of atherosclerosis to single or multiple arterial territories, the sCD40L levels were associated with the extent of atherosclerosis in the LE territory, and prior LE bypass surgery was associated with lower sCD40L levels among patients with LE atherosclerosis.

To the best of our knowledge, we present the first prospective study assessing sCD40L expression in single- and polyvascular atherosclerotic disease, including three major territories of atherosclerosis. sCD40L increased from controls to patients with isolated CAD and further to patients with combined CAD and LE disease (excluding those with prior revascularization). Investigation addressing inflammation in polyvascular atherosclerosis is relevant since it may have a distinct pathophysiology from that of single-vascular atherosclerosis and is a common clinical scenario associated with a higher risk morbidity and mortality [20,21,22]. Some inflammatory parameters have been associated with the presence of atherosclerosis in specific arterial territories and with atherosclerosis severity within each territory. However, data on the inflammatory signature in single- and multi-territorial atherosclerosis, with a prospective and systematic assessment of different territories, including the coronary, LE, and carotid artery territories, are very scarce [33]. Specifically, for sCD40L, which has a distinct mechanism of action compared with other inflammatory markers [5,6,7,8], such data are not available. Two studies reporting on the sCD40L levels in patients with stable atherosclerosis included a subgroup with polyvascular atherosclerosis but the sCD40L levels were not specifically reported in patients with single- and polyvascular disease [34,35]. In a study on patients with LE atherosclerosis, the sCD40L levels were specifically reported in a subgroup with coexistent CAD; however, not all patients with LE atherosclerosis were screened for CAD in this retrospective study, which limits the interpretation of the results [23]. In our study, the higher sCD40L levels in atherosclerosis of multiple (coronary and LE) territories suggest that sCD40L is a common denominator of the atherosclerosis expression.

The sCD40L levels were higher in combined CAD and LE atherosclerosis, but not in combined CAD and carotid artery disease, compared with CAD alone. We speculate that the regulation of the sCD40L levels may be mainly associated with the presence of CAD and the coexistence of carotid artery disease may not impact further on the sCD40L levels, on the contrary to LE disease. The local expression of inflammatory markers differs in carotid and femoral atherosclerotic plaques [36], and the stimulated LE iliac arteries may express more intensively CD40L in situ than the stimulated carotid arteries [37]. On the other hand, obstructive atherosclerosis of the LE could result in a higher degree of oxidative stress and inflammation compared with carotid artery disease considering the highly demanding LE muscles during physical effort and bilateral carotid blood supply to the cerebral territory [38]. For instance, the expression of miR-210, an adaptive microRNA to oxidative stress and inflammation, is altered in the presence of LE atherosclerosis but not in carotid artery disease [38]. This could explain the higher levels of the proinflammatory sCD40L in the presence of LE atherosclerosis compared with carotid atherosclerosis, in patients with CAD. Of note, the sCD40L levels were nonsignificantly higher in group 4 (atherosclerosis of the three arterial territories) compared with controls, corresponding to a trend consistent with the results of increased sCD40L levels in group 2 (atherosclerosis of the coronary and LE territories) compared with controls. On the other hand, group 4 presented similar sCD40L levels compared with group 1 (CAD alone). This finding is difficult to explain based on the extensive post hoc analysis performed. Nevertheless, group 4 presented a slightly better metabolic control compared with group 2, as reflected by nonsignificant lower fasting glycemia levels, higher HDL levels (which inhibit platelet activity through scavenger receptor B type I), and lower serum triglyceride levels, which may have contributed to lower sCD40L levels in group 4 [39,40,41,42,43]. We acknowledge that possible unmeasured confounders may have contributed to lower sCD40L levels in group 4.

The second main finding of this study was the association between the sCD40L levels and atherosclerosis extent within the LE territory. These results are consistent with the very few studies describing the sCD40L levels according to the severity of LE disease, assessed by the lesion length [23] or by an angiographic score based on the degree of luminal stenosis in each arterial segment [31]. For the carotid arteries, the association between the sCD40L levels and atherosclerosis severity is less well established [24].

Third, we observed that the sCD40L levels were lower in patients with prior LE bypass surgery. To our knowledge, this finding has not been reported. We speculate that the association between the proinflammatory sCD40L and atherosclerosis is bidirectional, with inflammation contributing to atherogenesis, as demonstrated in animal models [44], and with ischemia driven by atherosclerosis exacerbating inflammation [35]. In our sample, prior revascularization may have reduced ischemia, without modifying atherosclerosis burden of native arteries (since no endarterectomy was performed), which in turn may have reduced the release of reactive oxygen species, cytokines, and other inflammatory mediators, including sCD40L [26,45]. Consistently, the sCD40L levels were lower in patients with prior CABG compared with those without, among patients with CAD in this sample [12]. Immediately after CABG and LE surgical revascularization, an increase in the sCD40L levels has been reported, probably related to the surgical procedure [46,47]. The lower levels of sCD40L that we observed in the long term after LE revascularization is a novel finding, which may add to the knowledge on the pathophysiology of revascularization procedures and inflammation in stable atherosclerosis. Of note, although the number of participants with prior LE bypass surgery was small, this was a post hoc, hypothesis-generating analysis. Further studies are needed to confirm this hypothesis.

Finally, in the multivariate analysis, coexistent coronary and LE atherosclerosis and higher leukocyte count were independently associated with increased sCD40L levels, while prior CABG was associated with lower sCD40L levels. These data add consistency to the results and confirm the independent association between inflammation, disease extent, and ischemic burden.

This study has strengths that should be acknowledged. To our knowledge, we describe for the first time the variation of the sCD40L expression according to single- and multi-territorial atherosclerosis, including coronary, LE, and carotid atherosclerosis. The prospective nature of the study, the systematic screening of the three arterial territories and, importantly, the multivariate analysis carried out have contributed to higher consistency of our findings. In addition, analyses on the severity/extent of atherosclerosis in different territories added further consistency to the results. Finally, to the best of our knowledge, the association between prior LE surgical revascularization and lower sCD40L levels has not been reported.

Our study has some limitations. The sample may be of limited size; however, this study is pioneer in investigating the variation of sCD40L in single- and multi-territorial disease, and no data were available to support the sample size estimation. In this exploratory pilot study, the sample size was enough to detect differences in the sCD40L levels in atherosclerosis of single and multiple territories. On the other hand, as this is a single-center study, the results may not be applicable to different settings.

## 5. Conclusions

The sCD40L levels were higher in patients with atherosclerosis, particularly in those with polyvascular disease involving CAD and LE disease. The sCD40L levels increased with higher severity of LE atherosclerosis, assessed by the number of diseased segments, whereas prior LE surgical revascularization was associated with lower sCD40L levels. Our results provide insights into the pathophysiology of polyvascular atherosclerosis. In addition, sCD40L seems to be a promising noninvasive tool for refining the stratification of the systemic atherosclerotic burden and therefore could contribute to the tailoring of the primary prevention strategies. These fields deserve future research.

## Figures and Tables

**Table 1:** Characteristics of the participants according to the involved territories of atherosclerosis.

	Controls	Group 1	Group 2	Group 3	Group 4	*p*-Value
**Territories of atherosclerosis**	None	Coronary	Coronary + LE	Coronary + Carotid	Coronary + LE + Carotid	
*n*	26	20	18	12	18	
**Clinical characteristics**						
Age, years	59 (53–69)	65 (56–70)	67 (57–72)	59 (51–73)	69 (60–75)	0.079
Male, *n* (%)	23 (88.5)	18 (90.0)	16 (88.9)	10 (83.3)	17 (94.4)	0.912
Hypertension, *n* (%)	14 (53.8)	17 (85.0) ^1^	18 (100.0) ^1^	11 (91.7) ^1^	18 (100.0) ^1^	<0.001
Dyslipidemia, *n* (%)	18 (69.2)	19 (95.0) ^1^	18 (100.0) ^1^	11 (91.7)	17 (94.4) ^1^	0.010
Diabetes mellitus, *n* (%)	3 (11.5)	6 (30.0)	8 (44.4) ^1^	6 (50.0) ^1^	9 (50.0) ^1^	0.036
Smoking history, *n* (%)	6 (23.1)	9 (45.0)	12 (66.7) ^1^	4 (33.3)	12 (66.7) ^1^	0.014
LVEF > 50%, *n* (%)	26 (100.0)	20 (100.0)	18 (100.0)	12 (100.0)	18 (100.0)	–
Antiplatelet agent, *n* (%)	6 (23.1)	20 (100.0) ^1^	17 (94.4) ^1^	11 (91.7) ^1^	18 (100) ^1^	<0.001
Statin therapy, *n* (%)	13 (50.0)	18 (90.0) ^1^	16 (94.1) ^1^	11 (91.7) ^1^	16 (88.9) ^1^	0.001
**Laboratory parameters**						
Hemoglobin, g/dL	13.9 (12.9–15.0)	14.53 (10.0–15.1)	14.1 (13.2–14.6)	12.0 (11.4–13.4) ^2^	12.9 (12.1–14.2)	0.017
Leukocyte count, 10^9^/L	6.4 (1.7)	7.4 (1.9)	7.3 (1.7)	7.5 (2.2)	8.1 (1.7)	0.080
Neutrophil count, 10^9^/L	3.2 (2.5–4.8)	4.1 (3.4–5.2)	3.9 (3.4–4.8)	4.0 (3.4–6.7)	4.7 (3.6–6.0) ^1^	0.043
Lymphocyte count, 10^9^/L	1.9 (1.7–2.2)	2.1 (1.6–2.4)	2.1 (1.6–2.8)	1.7 (1.2–2.3)	2.2 (1.6–2.6)	0.401
Neutrophil/lymphocyte ratio	1.9 (0.7)	2.3 (1.1)	2.1 (1.0)	2.9 (1.1) ^1^	2.4 (1.0)	0.026
Platelet count, 10^9^/L	242 (191–274)	209 (176–269)	219 (195–264)	229 (137–251)	227 (203–263)	0.854
Fasting glycaemia, mg/dL	89 (80–98)	94 (86–129)	94 (83–125)	99 (84–157)	85 (75–123)	0.385
Percentage of glycosylated hemoglobin	5.6 (5.2–5.9)	5.9 (5.6–6.7)	5.9 (5.5–6.1)	5.8 (5.4–7.4)	5.9 (5.3–7.7)	0.185
Creatinine, mg/dL	0.8 (0.7–0.9)	0.9 (0.8–1.1)	0.8 (0.8–1.2)	0.9 (0.8–1.4)	1.1 (0.9–1.5) ^1^	0.002
Total cholesterol, mg/dL	186 (51)	164 (38)	172 (50)	153 (50)	173 (49)	0.329
LDL-cholesterol, mg/dL	99 (77–141)	95.0 (71–120)	106 (83–120)	65 (56–132)	117 (82–142)	0.297
HDL-cholesterol, mg/dL	51 (44–58)	35.0 (31–41) ^1^	35 (31–45) ^1^	40 (27–44) ^1^	40 (32–42) ^1^	<0.001
Triglycerides, mg/dL	106 (67–144)	142 (98–206)	115 (83–204)	100 (62–177)	117 (95–171)	0.423
C-reactive protein, mg/L	4.1 (2.0)	3.7 (1.4)	3.8 (1.1)	4.1 (2.3)	3.3 (2.0)	0.151
**Coronary artery disease**						
Nr. of vessels with obstructive disease *	0 (0–0)	3 (2–3) ^1^	3 (2–4) ^1^	3 (3–3) ^1^	3 (2–4) ^1^	<0.001
Nr. of obstructive lesions	0 (0–0)	4 (2–5) ^1^	4 (3–5) ^1^	4 (3–5) ^1^	4 (3–5) ^1^	<0.001
Gensini score	0 (0–0)	81 (41–97) ^1^	83 (42–118) ^1^	48 (40–116) ^1^	43 (36–87) ^1^	<0.001
Prior CABG, *n* (%)	0 (0.0)	7 (35.0) ^1^	7 (38.9) ^1^	6 (50.0) ^1^	3 (16.7) ^1^	0.002
**LE arterial disease**						
Bilateral disease, *n* (%)	0 (0.0)	0 (0.0)	10 (55.5) ^1,2,4,5^	0 (0.0)	15 (83.3) ^1–4^	<0.001
Any proximal lesion, *n* (%)	0 (0.0)	0 (0.0)	10 (55.5) ^1,2,4^	0 (0.0)	12 (66.7) ^1,2,4^	<0.001
Nr. of segments with obstructive disease	0 (0–0)	0 (0–0)	2 (1–4) ^1,2,4,5^	0 (0–0)	4 (3–5) ^1–4^	<0.001
Prior bypass surgery, *n* (%)	0 (0.0)	0 (0.0)	2 (11.1) ^1,2,4^	0 (0.0)	6 (33.3) ^1,2,4^	<0.001
**Carotid artery disease**						
Bilateral disease, *n* (%)	0 (0.0)	0 (0.0)	0 (0.0)	3 (25.0) ^1–3^	8 (44.4) ^1–3^	<0.001
Mean IMT, mm	0.68 (0.11)	0.67 (0.14)	0.76 (0.06)	-	-	0.296
Maximal IMT, mm	0.80 (0.13)	0.86 (0.18)	0.94 (0.09)	-	-	0.105

Categorical variables are expressed as frequency (percentage) and continuous variables as mean (standard deviation) or median (interquartile range). CABG—coronary artery bypass grafting; HDL—high-density lipoproteins; IMT—intima–media thickness; LDL—low-density lipoproteins; LE—lower extremity; LVEF—left ventricular ejection fraction; Nr.—number. ^1^ *p*-Value < 0.05 vs. controls; ^2^ *p*-Value < 0.05 vs. group 1; ^3^ *p*-Value < 0.05 vs. group 2; ^4^ *p*-Value < 0.05 vs. group 3; ^5^ *p*-Value < 0.05 vs. group 4; * for the assessment of the number of vessels with obstructive disease, the left main, left anterior descending, circumflex, and right coronary arteries were considered separately, with a total score ranging from 0 to 4.

**Table 2:** Independent predictors of the soluble CD40 ligand levels by multivariate linear regression analysis.

Predictors	β	95% CI	*p*-Value
Coexistent coronary and lower extremity atherosclerosis	2.512	1.054 to 3.970	0.001
Prior coronary artery bypass grafting	−1.758	−3.159 to −0.357	0.015
Leukocyte count	0.417	0.102 to 0.732	0.010

95% CI—95% confidence interval.

## Data Availability

The data presented in this study are available on request from the corresponding author. The data are not publicly available due to personal data protection.

## Supplementary Materials

The following are available online at https://www.mdpi.com/1010-660X/57/1/39/s1, Table S1: Association of soluble CD40 ligand levels with clinical characteristics, laboratory results, and atherosclerosis data, Table S2: Characteristics of patients with lower extremity atherosclerosis with and without prior lower extremity bypass surgery, Table S3: Association of soluble CD40 ligand levels with clinical characteristics and laboratory results, stratified by the study group.
