# Supplementary material for: The Proinflammatory Soluble CD40 Ligand Is Associated with the Systemic Extent of Stable Atherosclerosis
Source: Medicina (Kaunas). 2021 Jan 4;57(1):39. doi: 10.3390/medicina57010039 (PMC7824733; doi:10.3390/medicina57010039)
Supplement: Supplementary file 1 [file medicina-57-00039-s001.pdf]

Table S1. Association of soluble CD40 ligand levels with clinical characteristics, laboratory results, and atherosclerosis data.

		sCD40L, ng/mL	*p*-Value
**Clinical data ***			
Age, years [1]		r = -0.085	0.419
Sex [2]	Male	4.2 (2.7–8.8)	0.876
	Female	5.1 (2.9–7.2)	
Hypertension [2]	No	4.3 (2.9–6.0)	0.555
	Yes	5.1 (2.9–7.6)	
Dyslipidemia [2]	No	5.2 (2.7–7.0)	0.953
	Yes	5.0 (2.9–7.6)	
Diabetes mellitus [2]	No	5.0 (3.2–7.3)	0.555
	Yes	4.8 (2.3–7.6)	
Smoking history [2]	No	4.2 (2.8–7.1)	0.363
	Yes	5.9 (2.9–7.8)	
**Laboratory data ***			
Hemoglobin, g/dL [1]		r = 0.123	0.243
Leukocyte count, 10^9/L [1]		r = 0.301	0.004
Neutrophil count, 10^9/L [1]		r = 0.219	0.037
Lymphocyte count, 10^9/L [1]		r = 0.292	0.005
Neutrophil/lymphocyte ratio [1]		r = -0.041	0.704
Platelet count, 10^9/L [1]		r = 0.223	0.035
Fasting glycaemia, mg/dL [1]		r = 0.114	0.284
Percentage of glycosylated hemoglobin [1]		r = 0.136	0.217
Creatinine, mg/dL [1]		r = 0.118	0.258
Total cholesterol, mg/dL [1]		r = -0.017	0.874
LDL-cholesterol, mg/dL [1]		r = 0.014	0.897
HDL-cholesterol, mg/dL [1]		r = -0.222	0.036
Triglycerides, mg/dL [1]		r = 0.039	0.717
C-reactive protein, mg/L [1]		r = 0.095	0.388
**Atherosclerosis data**			
**Coronary artery disease ***			
Nr. of vessels with obstructive disease [1]		r = 0.285	0.006
Nr. of obstructive lesions [1]		r = 0.238	0.022
Gensini score [1]		r = 0.279	0.007
Prior coronary artery bypass grafting [2]	No	6.8 (3.5)	0.001
	Yes	4.3 (2.1)	
**LE arterial disease**			
Nr. of sides affected [1]	One	6.0 (4.0)	0.822
	Two	5.8 (3.0)	
Any proximal lesion [2]	No	7.3 (3.7)	0.101
	Yes	5.3 (3.0)	
Nr. of segments with obstructive disease [1]		r = 0.157	0.147
Prior bypass surgery [2]	No	6.8 (3.5)	0.036
	Yes	3.9 (2.5)	
**Carotid artery disease**			
Nr. of sides affected [2]	One	5.2 (3.1)	0.083
	Two	5.7 (2.7)	
Mean intima–media thickness, mm [1]		r = -0.200	0.215
Maximal intima–media thickness, mm [1]		r = -0.169	0.271

[1] Correlations between soluble CD40 ligand levels and continuous variables were tested and the correlation coefficient (r) is presented for each; [2] soluble CD40 ligand levels were compared between groups for categorical variables and are expressed as mean (standard deviation) or median (interquartile range). Each tested association under the heading of the respective territory of disease (coronary, lower extremity, and carotid) included only patients with obstructive disease of the corresponding territory, with the exception of intima–media thickness, which only included participants without obstructive carotid artery disease. HDL – high-density lipoproteins; LDL – low-density lipoproteins; LE – lower extremity; Nr. – number; sCD40L – soluble CD40 ligand. * Pereira-da-Silva, T.; Napoleao, P.; Pinheiro, T.; Selas, M.; Silva, F.;

Ferreira, R.C.; Carmo, M.M. Inflammation is associated with the presence and severity of chronic coronary syndrome through soluble CD40 ligand. *Am J Cardiovasc Dis* **2020**, *10*, 329-339.

Table S2. Characteristics of patients with lower extremity atherosclerosis with and without prior lower extremity bypass surgery.

	**No prior LE bypass surgery** (n = 28)	**Prior LE bypass surgery** (n = 8)	p-Value
**Clinical characteristics**			
Age, years	67 (8.5)	67 (7.0)	0.887
Male, n (%)	26 (92.9)	7 (87.5)	0.541
Hypertension, n (%)	28 (100.0)	8 (100.0)	–
Dyslipidemia, n (%)	27 (96.4)	8 (100.0)	1.000
Diabetes mellitus, n (%)	12 (42.9)	5 (62.5)	0.281
Smoking history, n (%)	18 (64.3)	6 (75.0)	0.691
LVEF > 50%, n (%)	28 (100.0)	8 (100.0)	–
Antiplatelet agent, n (%)	27 (96.4)	8 (100.0)	1.000
Statin therapy, n (%)	25 (89.3)	7 (87.5)	0.553
**Laboratory parameters**			
Hemoglobin, g/dL	13.8 (1.5)	12.9 (1.7)	0.193
Leukocyte count, 10^9/L	7.8 (1.6)	7.1 (2.1)	0.288
Neutrophil count, 10^9/L	4.1 (3.4–5.5)	3.9 (2.5–5.9)	0.762
Lymphocyte count, 10^9/L	2.4 (0.8)	1.8 (0.5)	0.101
Neutrophil/lymphocyte ratio	2.1 (0.9)	2.6 (1.3)	0.304
Platelet count, 10^9/L	225 (44)	235 (49)	0.620
Fasting glycaemia, mg/dL	87 (79–121)	106 (85–160)	0.229
Percentage of glycosylated hemoglobin	5.9 (5.5–7.1)	5.5 (5.0–9.6)	0.439
Creatinine, mg/dL	0.9 (0.8–1.3)	1.3 (1.0–1.6)	0.083
Total cholesterol, mg/dL	172 (46)	174 (63)	0.918
LDL-cholesterol, mg/dL	109 (38)	111 (44)	0.610
HDL-cholesterol, mg/dL	37 (30–42)	35 (34–46)	0.793
Triglycerides, mg/dL	117 (89–173)	163 (87–203)	0.558
C-reactive protein, mg/L	3.4 (1.9)	3.8 (2.1)	0.611
**Coronary artery disease**			
Presence of coronary artery disease, n (%)	28 (100.0)	8 (100.0)	–
Nr. of vessels with obstructive disease *	3 (3–4)	3 (2-4)	0.284
Nr. of obstructive lesions	4 (3–5)	4 (3–5)	0.668
Gensini score	78 (39–116)	49 (24–46)	0.101
Prior CABG, n (%)	7 (25.0)	3 (37.5)	0.658
**LE arterial disease**			
Bilateral disease, n (%)	18 (64.3)	7 (87.5)	0.201
Any proximal lesion, n (%)	15 (53.4)	7 (87.5)	0.218
Nr. of segments with obstructive disease	2.8 (1.6)	4.1 (1.8)	0.064
Time elapsed from bypass surgery, years	–	4 (2–9)	–
**Carotid artery disease**			
Presence of carotid artery disease, n (%)	12 (42.9)	6 (75.9)	0.638
Bilateral disease, n (%)	6 (21.4)	2 (25.0)	0.207
Mean IMT, mm	0.77 (0.06)	0.75 (0.07)	0.463
Maximal IMT, mm	0.95 (0.09)	0.92 (0.10)	0.457

Categorical variables are expressed as frequency (percentage) and continuous variables as mean (standard deviation) or median (interquartile range). CABG – coronary artery bypass grafting; HDL – high-density lipoproteins; IMT – intima–media thickness; LDL – low-density lipoproteins; LE – lower extremity; LVEF – left ventricular ejection fraction; Nr. – number. * For the assessment of the number of vessels with obstructive disease, the left main, left anterior descending, circumflex, and right coronary arteries were considered separately, with a total score ranging from 0 to 4.

Table S3. Association of soluble CD40 ligand levels with clinical characteristics and laboratory results, stratified by the study group.

		Controls		Group 1		Group 2		Group 3		Group 4	
**Territories of atherosclerosis**		None		Coronary		Coronary + LE		Coronary + Carotid		Coronary + LE + Carotid	
		sCD40L, ng/mL	*p*-Value	sCD40L, ng/mL	*p*-Value	sCD40L, ng/mL	*p*-Value	sCD40L, ng/mL	*p*-Value	sCD40L, ng/mL	*p*-Value
**Clinical characteristics**											
Age, years [1]		r=0.040	0.850	r=-0.148	0.533	r=-0.342	0.165	r=-0.014	0.965	r=-0.253	0.312
Sex [2]	Male	3.9 (2.8–5.2)	0.280	6.8 (3.1–7.9)	0.674	7.5 (2.9–11.1)	0.574	4.0 (2.2–7.9)	0.364	5.2 (2.7–6.6)	–
	Female	2.8 (2.5–3.0)		4.2 (4.2–4.2)		5.9 (2.1–9.8)		8.0 (5.3–10.7)		7.7	
Hypertension [2]	No	3.7 (2.7–5.2)	0.852	6.6 (3.3–9.2)	0.416	–	–	9.9	–	–	–
	Yes	3.9 (2.8–5.6)		5.1 (3.1–7.7)		7.5 (2.6–10.4)		4.3 (2.4–7.2)		5.5 (3.0–7.2)	
Dyslipidemia [2]	No	4.4 (2.7–5.4)	0.798	7.5	–	–	–	9.9	–	7.0	–
	Yes	3.6 (2.8–5.1)		5.1 (3.2–7.9)		7.5 (2.6–10.4)		4.3 (2.4–7.2)		5.2 (2.7–7.0)	
Diabetes mellitus [2]	No	4.0 (2.8–5.2)	0.128	5.8 (3.5–7.6)	0.779	9.5 (5.0–12.7)	0.173	3.8 (1.9–10.2)	0.394	5.2 (3.2–6.9)	1.000
	Yes	4.2 (2.1–7.1)		5.1 (2.3–8.8)		4.6 (2.2–9.5)		5.6 (3.8–8.7)		5.8 (2.0–7.4)	
Smoking history [2]	No	3.9 (2.7–5.2)	0.733	4.2 (3.2–8.5)	0.941	9.3 (2.1–10.6)	0.964	4.8 (2.0–6.9)	0.368	5.5 (2.9–7.9)	0.892
	Yes	3.0 (2.8–5.3)		7.0 (3.3–7.7)		6.1 (2.9–11.1)		7.2 (3.8–12.6)		5.5 (2.5–6.8)	
**Laboratory parameters**											
Hemoglobin, g/dL [1]		r=0.132	0.528	r=0.312	0.181	r=0.280	0.261	r=0.141	0.662	r=-0.242	0.349
Leukocyte count, 10^9/L [1]		r=-0.468	0.021	r=0.264	0.290	r=0.625	0.006	r=0.617	0.033	r=-0.034	0.897
Neutrophil count, 10^9/L [1]		r=-0.447	0.025	r=0.184	0.451	r=0.406	0.094	r=0.577	0.050	r=0.006	0.980
Lymphocyte count, 10^9/L [1]		r=-0.063	0.769	r=0.373	0.116	r=0.381	0.119	r=0.377	0.227	r=0.000	0.999
Neutrophil/lymphocyte ratio [1]		r=-0.446	0.022	r=-0.201	0.423	r=-0.016	0.949	r=0.145	0.652	r=-0.159	0.542
Platelet count, 10^9/L [1]		r=-0.091	0.666	r=0.439	0.068	r=0.469	0.050	r=0.400	0.197	r=-0.179	0.491
Fasting glycaemia, mg/dL [1]		r=0.233	0.262	r=0.202	0.407	r=-0.137	0.587	r=-0.108	0.737	r=0.416	0.096
Percentage of glycosylated hemoglobin [1]		r=0.188	0.414	r=0.306	0.232	r=-0.041	0.875	r=-0.250	0.434	r=0.369	0.145
Creatinine, mg/dL [1]		r=-0.316	0.124	r=0.011	0.963	r=-0.153	0.544	r=0.707	0.010	r=-0.033	0.895
Total cholesterol, mg/dL [1]		r=-0.061	0.776	r=0.135	0.571	r=0.183	0.468	r=0.077	0.812	r=-0.376	0.137
LDL-cholesterol, mg/dL [1]		r=0.020	0.925	r=0.043	0.856	r=0.245	0.328	r=0.152	0.636	r=-0.383	0.129
HDL-cholesterol, mg/dL [1]		r=-0.229	0.283	r=-0.293	0.223	r=0.050	0.843	r=-0.325	0.302	r=0.175	0.501
Triglycerides, mg/dL [1]		r=-0.218	0.306	r=0.279	0.248	r=0.079	0.755	r=-0.174	0.588	r=-0.392	0.119
C-reactive protein, mg/L [1]		r=0.184	0.411	r=0.300	0.227	r=-0.135	0.618	r=0.331	0.293	r=-0.007	0.978

[1] Correlations between soluble CD40 ligand levels and continuous variables were tested and the correlation coefficient (r) is presented for each; [2] soluble CD40 ligand levels were compared between groups for categorical variables and are expressed as mean (standard deviation) or median (interquartile range). HDL – high-density lipoproteins; LDL – low-density lipoproteins; sCD40L – soluble CD40 ligand.
